# Smoking induces increased apoptosis in osteoblasts: changes in bone matrix organic components

**DOI:** 10.1038/s41598-023-33965-8

**Published:** 2023-04-28

**Authors:** Júlia Benini Kohler, Alex Ferreira da Silva, Walleson Alves Farias, Barbara Fialho Carvalho Sampaio, Marco Aurélio Silveiro Neves, Leandro Gregorut Lima, Juliana Dias Lourenço, Alyne Riani Moreira, Alexandre Póvoa Barbosa, Iolanda de Fátima Lopes Calvo Tibério, Walcy Rosolia Teodoro, Fernanda Degobbi Tenorio Quirino dos Santos Lopes

**Affiliations:** 1 Department of Clinical Medicine, Laboratory of Experimental Therapeutics (LIM-20), School of Medicine, University of Sao Paulo, São Paulo, Brazil; 2 Rheumatology Division of the Hospital das Clinicas FMUSP, School of Medicine, University of Sao Paulo, São Paulo, Brazil; 3 Department of Medicine, School of Medicine, University of Sao Paulo, Av. Dr. Arnaldo 455-Room 1220, São Paulo, SP 01246-903 Brazil

**Keywords:** Osteoimmunology, Musculoskeletal system

## Abstract

Clinical studies demonstrate the impact of smoking on bone tissue fragility and higher incidence of fractures. However, it is not totally understood which physiological mechanisms could be involved in these events. Previously, we showed important changes in bone tissue components in experimental model of cigarette smoke (CS) exposure. CS exposure induces worsening in bone mineralization and a decrease in collagen type I deposition, leading to bone fragility. Considering that the majority of clinical studies described bone structural changes by radiographic images, in this study we performed analyses “in situ” using tissue samples from smokers, former smokers and non-smokers to better understand how the increase in inflammatory mediators induced by smoking exposure could interfere in bone cells activity leading bone structural changes. We observed increased levels of IL-1β, IL-6 and TNF-α in bone tissue homogenates with a concomitant increase in osteoblast apoptosis in smokers and former smokers compared with non-smokers. Histological changes in both smokers and former smokers were characterized by reduction in collagen type I. Only in smokers, it was observed decrease in trabecular area, suggesting increased bone resorption and increase in collagen type V. These results showed that osteoblasts apoptosis in association with increased bone resorption leads bone structural changes in smokers.

## Introduction

Smoking remains the global leader in causes of preventable deaths worldwide^1^. In bone, its influence in the impairment of homeostasis has been recognized^2–6^. These effects are attributed to their gaseous and particular compounds that induce chronic airway inflammation and tissue destruction^7–9^.

Bone is an organized and specialized connective tissue composed by cells and extracellular matrix components (organic and inorganic). The inorganic constituent of bone matrix is mainly composed by crystalline hydroxyapatite-[Ca_3_(PO_4_)_2_]_3_ Ca(OH)_2_, and although the organic component contains around 20 proteins, the type I collagen is the most prevalent^10^. There are two large groups of specialized cells in bone tissue that are responsible for its homeostasis: osteoblastic lineage cells (osteoblasts, osteocytes and lining) and bone resorption cells (osteoclasts)^11^. The osteoblasts are recognized by the extracellular matrix components production, whereas the osteoclasts are the only cells capable of resorbing the mineralized bone matrix^12^. Experimental and in vitro studies have demonstrated that smoking induces bone loss and reduces bone mineral density^13–15^ and these effects are mainly attributed to the stimulation of osteoclastogenesis and inhibition of osteoblastogenesis^13,16^.

Previously, we showed important changes in bone tissue components in experimental model of cigarette smoke (CS) exposure. CS exposure induces worsening in bone mineralization with a concomitant decrease of collagen type I deposition, leading to bone fragility^13^. In addition, we performed temporal analysis in mice exposed to CS for 1, 3 and 6 months. We observed a progressive reduction of type I collagen and reduction in COL1A1 gene expression^17^. Moreover, we demonstrated that there was an increase in osteoclastogenesis that culminate in reduction of bone volume, trabecular thickness and the number of trabeculae after 6 months of CS exposure^18,19^.

Despite of clinical evidence of the deleterious effects of smoking in bone, mostly of these findings are based in bone images and clinical parameters and there is a lack of studies in bone tissue samples which difficult to characterize its impact in bone matrix components as well as which inflammatory mediators could participate in these events.

To better understand the effects of smoking in bone tissue, we performed analyses in tissue samples from smokers, former smokers and non-smokers. We described the impact of smoking in both bone structural and inflammatory mediators release.

## Results

104 patients were collected and characteristics of the patients were analyzed: age, BMI, gender, pack years, use of ICS, alcohol intake and physical exercise are presented at Table 1.

**Table 1:** Data are presented as the mean ± standard deviation. *p = 0.002 compared with former smokers.

	Control (n = 40)	Former smokers (n = 31)	Smokers (n = 33)
Age (years)	57.41 ± 15	61,323 ± 12	60,545 ± 11
BMI (kg/m^2^)	27.77 ± 5	26.21 ± 4	27.50 ± 5
Male/female (n)	20/20	15/16	22/11
Pack years (years)	–	6956 ± 7946	19,230 ± 21,445*
Use of ICS	3/40	1/31	0/33
Alcohol intake (yes)/(no)	3/40	3/31	3/33
Physical exercise (yes)/(no)	8/40	7/31	4/33

Data of smoking cessation years were analyzed and compared for better understanding of Former Smokers group (Table 2).

**Table 2:** Smoking cessation patients in years.

Smoking cessation Former Smokers (n = 32)	%
1–2 years	12.5
2–5 years	6.25
5–10 years	12.5
Above 10 years	68.75

### Trabecular volume analyses

**Figure 1:**
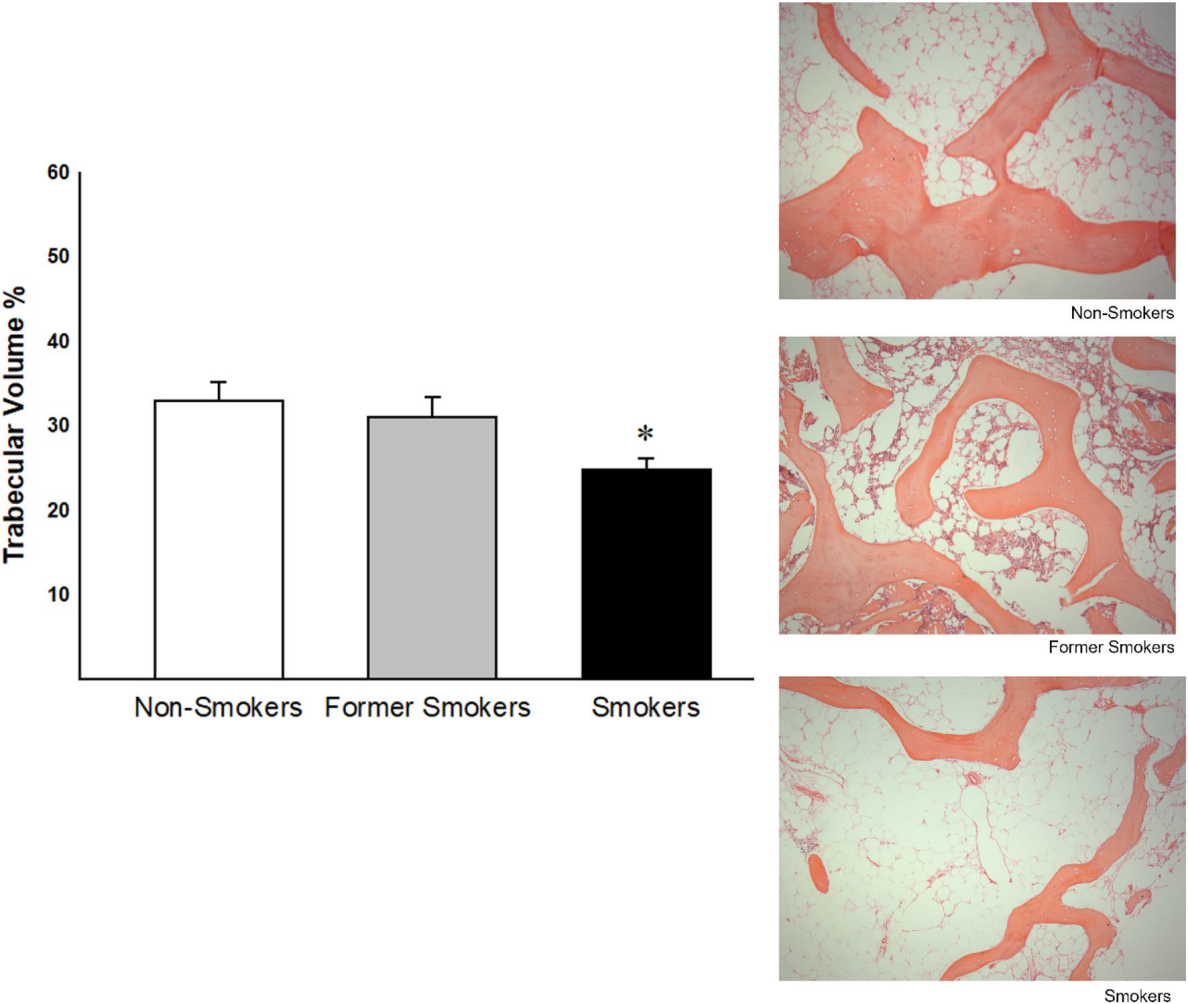
Trabecular volume in non-smokers, former smokers and smokers (n = 19, n = 22, n = 22 respectively). Significant differences were found in smokers group compared with non-smokers (*p = 0.023 Dunn's test). Photomicrographs of trabeculae in bone tissue from femoral head-neck transition area. Results were expressed as means ± SE (×100 magnification).

Trabecular volume analysis revealed a significant decrease in smokers compared with non-smokers (Fig. 1).

### Percentage Type I collagen

**Figure 2:**
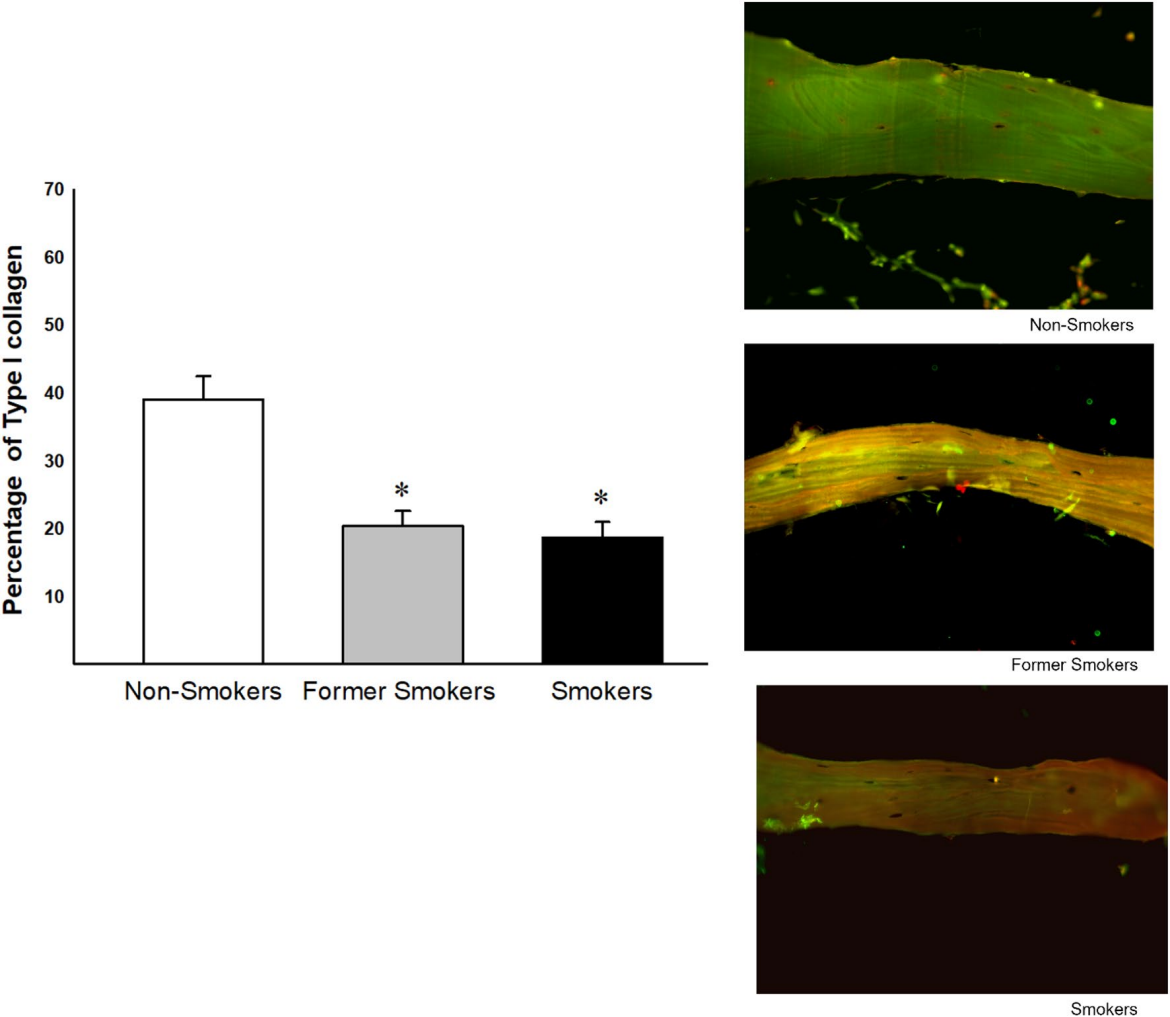
Percentage of Type I collagen in non-smokers, former smokers and smokers (n = 19, n = 22, n = 22 respectively). Significant differences were found in former smokers, and smokers compared with non-smokers (*p =  < 0.001, Dunn's Test). Photomicrographs of Collagen Type I in bone tissue from femoral head-neck transition area. Values ​​were expressed as mean ± SE (×400 magnification).

The percentage of Type I collagen was decreased in Former Smokers and Smokers compared with Non-smokers group (Fig. 2).

### Percentage Type V collagen

**Figure 3:**
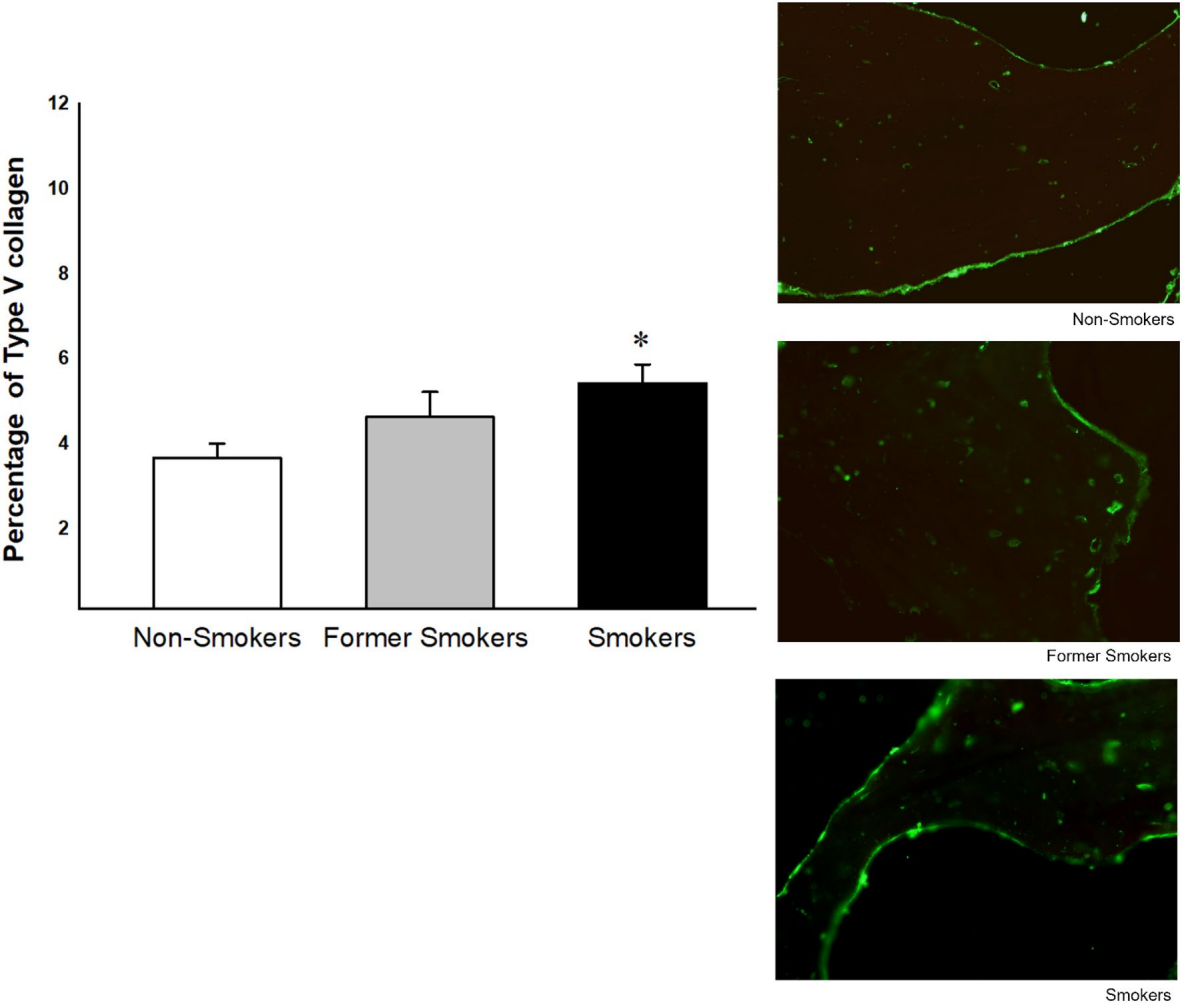
Percentage of Type V collagen in non-smokers, former smokers and smokers (n = 19, n = 22, n = 22 respectively). Significant differences were found in smokers compared to non-smokers (*p =  < 0.047, Dunn's Test). Photomicrographs of collagen Type I in bone tissue from femoral head-neck transition area. Values were expressed as mean ± SE (×400 magnification).

The percentage of Type V collagen was increased in smokers compared with non-smokers group (Fig. 3).

### Cytokine analysis in bone samples

**Figure 4:**
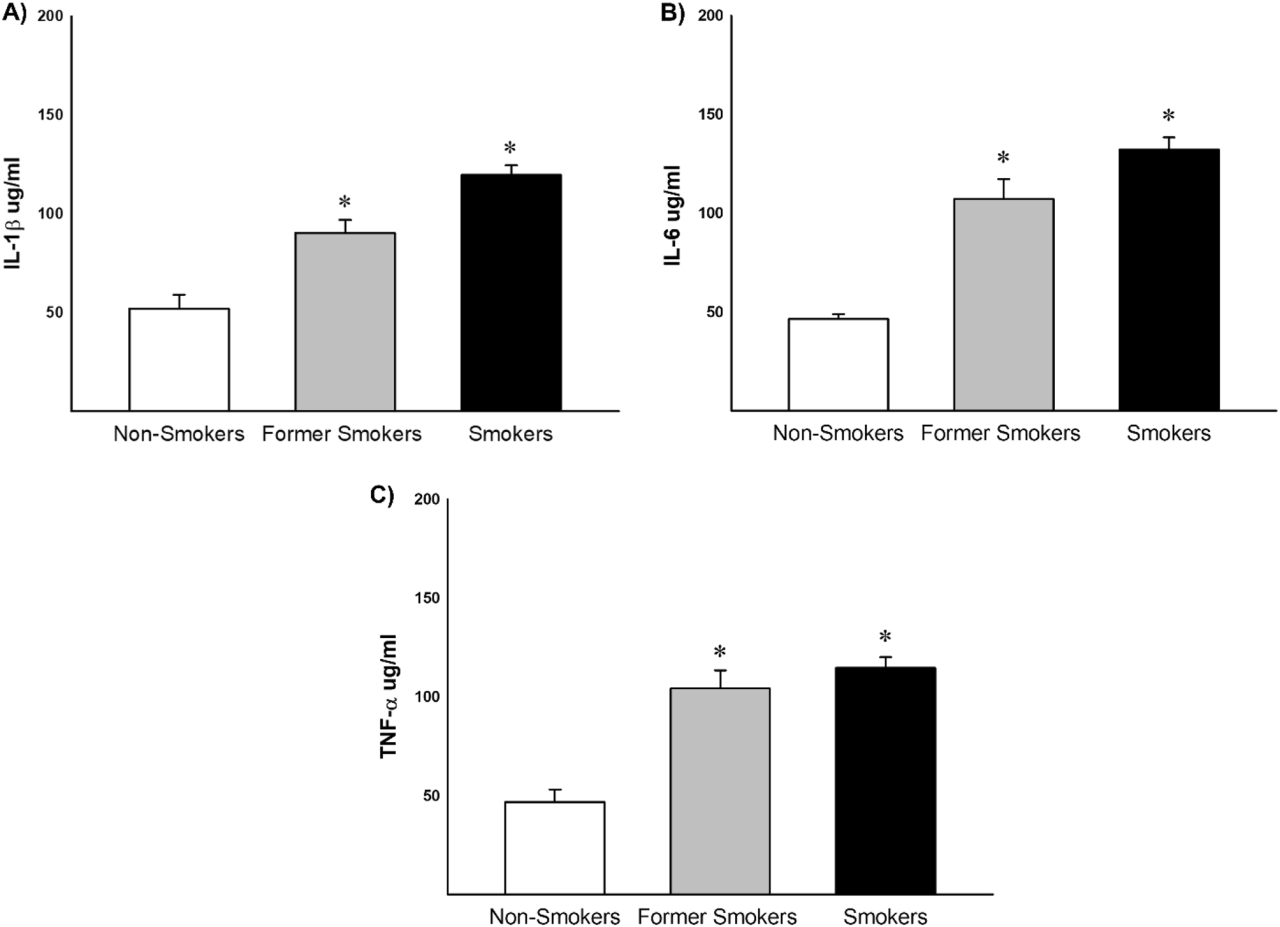
Expression of IL-1β, IL-6 and TNF-α in bone homogenates of non-smokers, former smokers and smokers (**A**) n = 13, n = 16, n = 25 respectively), (**B**) n = 16, n = 18, n = 25 IL-6 respectively) and (**C**) n = 16, n = 22, n = 25 respectively). Significant increased were found in former smokers and smokers compared with non-smokers in IL-1β (*p =  < 0.001, Dunn's Test), IL-6 (*p = 0.001, Dunn's Test) and TNF-α (*p =  < 0.001, Dunn's Test). Values were expressed as mean ± SE.

Cytokine IL-1β, IL-6 and TNF-α were evaluated in bone samples by ELISA technique. The same inflammatory profile was observed in the three analyzes we observed increased results in former smokers and smokers compared with non-smokers (Fig. 4).

**Figure 5:**
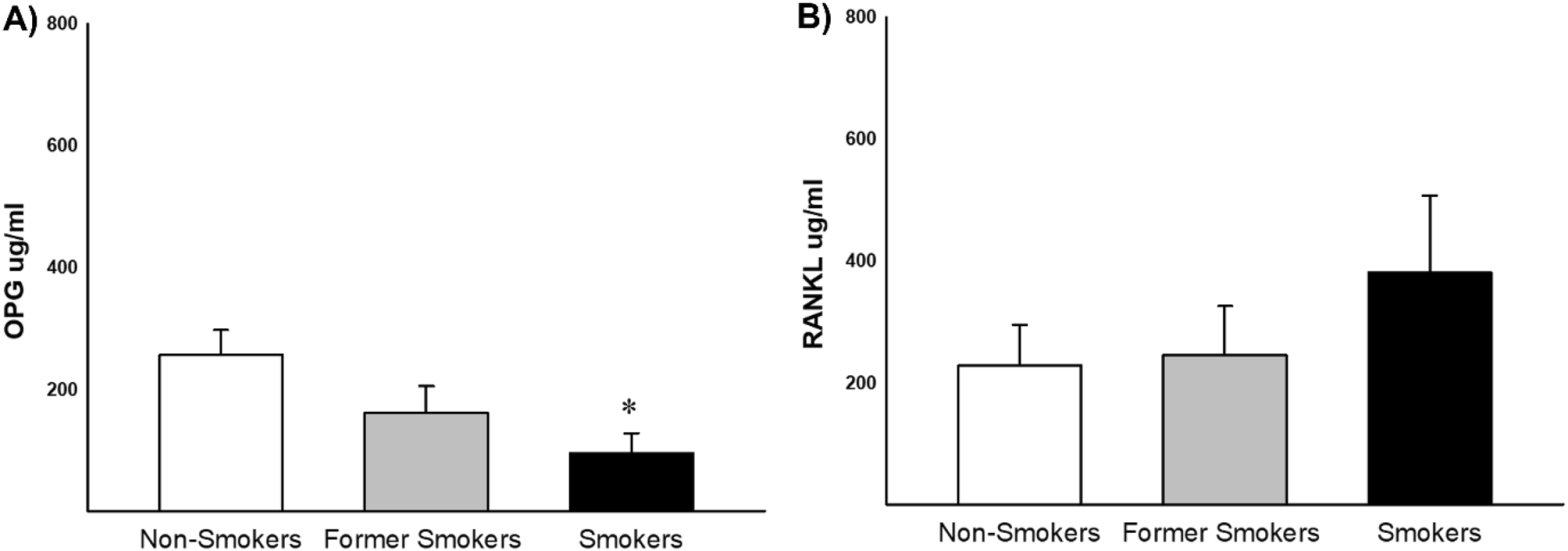
Expression of OPG and RANKL in bone homogenate of non-smokers, former smokers and smokers (**A**) n = 24, n = 24, n = 21 respectively), (**B**) n = 8, n = 13, n = 16 respectively). Significant decrease was found in smokers compared with non-smokers group in OPG (*p = 0.014, Dunn's Test). Values were expressed the mean ± SE.

Protein quantification of OPG and RANKL were evaluated in bone samples by ELISA technique, and we observed decrease results in Smokers compared with Non-smokers (Fig. 5A). There was no statistical difference in RANKL evaluation (Fig. 5B).

### Immunohistochemistry for osteoblasts apoptosis Caspase-3

**Figure 6:**
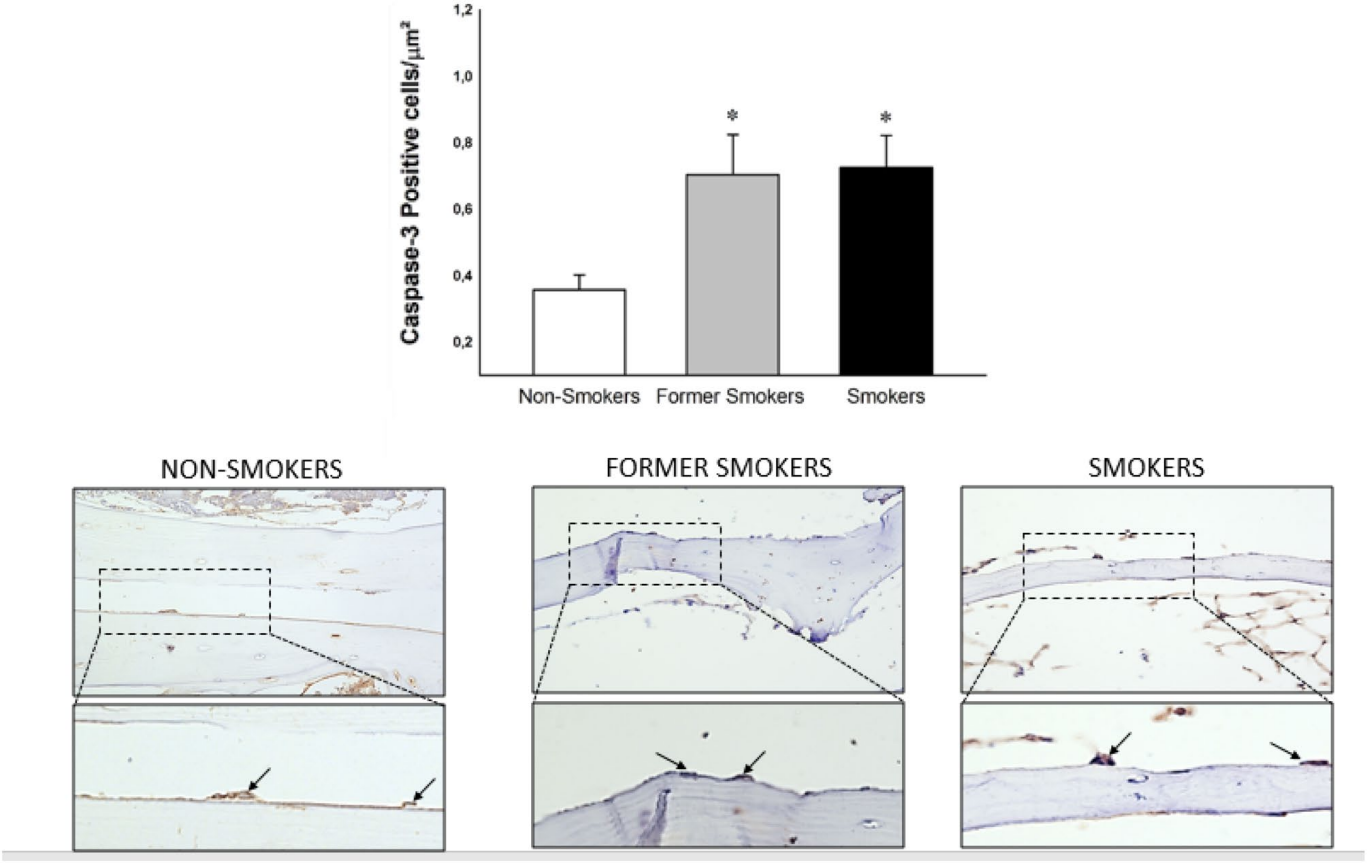
The density of Caspase-3 positive cells for non-smokers, former smokers and smokers (n = 12, n = 12, n = 10 respectively). Significant increase was found in former smokers and smokers compared with non-smokers group (*p = 0.005, Dunn's Test). Photomicrographs of Caspase-3 positive cells in bone tissue from femoral head-neck transition area. Results were expressed as the means ± SE (×400 magnification).

The density of Caspase-3 positive cells was increased in former smokers and smokers group compared with non-smokers (Fig. 6).

## Methods

### Study subjects and case information

All participants included in this research provided written informed consent prior to participation and all methods were carried out in accordance with relevant guidelines and regulations of Helsinki declaration of 1964 and its amendments^20^. This study was approved by the local research ethics committee (School of Medicine, University of Sao Paulo Ethics Committee, protocol (3.120.116).

For this study, 104 individuals who underwent total hip arthroplasty from February 2019 to March 2022, from the Hospital Municipal de São José dos Campos were included. This study used femoral head of patients who were previously evaluated and indicated for total hip arthroplasty surgery and there was no risk to the patient's life since the collections were previously indicated to patients with hip arthrosis and all the tissues used in this work would be discarded. It was included patients with primary osteoarthritis from degenerative and mechanical causes. We did not include patients with post-traumatic osteoarthritis, sequelae of childhood diseases, hip dysplasia and vascular pathologies. Also, it was not included patients with previous fracture due osteoporosis like proximal humerus, wrist and hip. Samples of arthroplasties performed for fractures of the proximal femur were not collected, since it is a result of osteoporotic bone.

In addition to the orthopedic evaluation, all patients were clinically evaluated preoperatively. The clinical questionnaires of these evaluations generated the database that defined the inclusion and exclusion criteria in the research, regarding comorbidities, associated pathologies and regular use of medication.

Patients with Diabetes Mellitus, systemic arterial hypertension and dyslipidemia were included in the research if they were clinically compensated and in use of their medications regularly. We excluded patients with oncological diagnoses, autoimmune diseases, vascular injuries under treatment, regular use of anticoagulants and anticonvulsants, and those in use of medication for the treatment of osteoporosis (bisphosphonates or hormone replacement).

Demographic data, medical history and smoking habits were collected. All patients included in this study signed an informed consent form (ICF).

Patients were divided into three groups: smokers: patients who are active cigarette smoking (n = 33), former smokers: patients who quit cigarette smoking for at least 1 year (n = 31) and non-smokers: patients who have never had exposure to cigarette smoke (n = 40). The exclusion criteria are autoimmune or/and oncological.

The dataset used during the current study is available from the corresponding author upon reasonable request.

### Material collection

**Figure 7:**
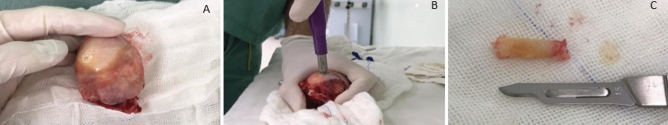
Photomicrographs of femoral head (**A**), bone tissue sample collection from the lateral aspect of subcapital region of the femoral neck with a 5.5 mm trephine (**B**) and bone tissue sample (**C**).

Bone tissue samples were collected from the lateral aspect of the subcapital region of the femoral head (femoral head-neck transition area) with a 5.5 mm trephine (Fig. 7).

Samples were divided into three different analyses: samples stored at – 70 °C and pulverized for ELISA and PCR and samples stored in room temperature in nitric acid for histology and morphometry.

### Histology

Bone tissue was collected and decalcified with a 7% nitric acid solution for 48 h, washed in running water for 10 min, in a running water bath and immersed in 70% alcohol. The samples were processed through the HistoCore PEARL Tissue Processor and later the tissue was embedded in paraffin. 4 µm thick sections were made with a space of 50 µm between them for use in histological staining techniques.

### Trabecular volume analysis

The slides were stained with hematoxylin and eosin (HE) and subsequently were analyzed under microscope and quantified by an image analysis system. The system that was used consisted of a Sony CCD camera, coupled to an microscope, from which the images were viewed on the monitor. Through a digital system inserted in a computer (Pentium 4), the images were processed by Image-Pro Plus 6.0 software. The samples were analyzed blindly, with 5–10 fields being randomly selected in the trabecular region at a magnification of 100×. The total area of tissue analyzed in each field was measured by the Image-Pro Plus 6.0 software. The trabecular volume area was divided by the total area of tissue analyzed and the result was expressed as a percentage.

### Collagen I and V measurements

For immunofluorescence of Type I and V collagen, 4 µm thick sections of bone tissue were adhered to a slide treated with a Poly-l-Lysine adhesive and high strength epoxy surface to facilitate the process of hydrophobic and color marking of bone tissue. Cuts were made through temperature slides to heated xylene, at 60 °C, for 30 min, and two 10-min baths in ambient xylene. Rehydration was carried out by successful ethylic wash for minutes, 1 min of crescents, followed by washing in running water, for 10 baths in running water and PBS 15 running, pH 7.5. For exposure and recovery of antigenic sites, sections were selected for digestion with bovine pepsin at a concentration of 8 mg/500 µl in 0.5N acetic acid, for 30 min at 37 °C. At the end of this incubation, the sections were included in a wash cycle with PBS, three times for 10 min, and the nonspecific sites were blocked with 5% bovine serum albumin (BSA) in PBS, for 30 min, at room temperature. Slides were incubated overnight with rabbit polyclonal anti-collagen type I (1:100; Rockland) anti-collagen type V (1:100; Rockland), diluted in PBS solution. After this, sections were washed in PBS with 0.05% Tween20 and incubated for 1 h with ALEXA 488 goat anti-rabbit IgG (Invitrogen, Life Technologies) diluted 1:200 in Evans Blue. Finally, the slides were washed again five times with Tween 20, mounted with buffered glycerin solution and glycerin in fluorescence buffer.

Slides were analyzed by the same image analysis system used for trabecular area analysis. The samples were analyzed blindly, with 5–10 fields being randomly selected in the trabecular region at a magnification of 400×. The total area of tissue analyzed in each field was measured by the Image-Pro Plus 6.0 software. The collagen present in the acquired fields was evaluated by selecting the fluorescent green hue, corresponding to each labeled collagen. The immunostained area was divided by the total area of tissue analyzed and the result was expressed as a percentage.

### Cytokine analysis

Levels of IL-1β, IL-6, TNF-α, OPG and RANKL were analyzed by enzyme-linked immunosorbent assay (ELISA). As described by Kohler^21^ the samples were evaluated by in pulverized bone using microplates for each cytokine sensitized with specific monoclonal antibodies. After washing and distribution of the samples, specific antibodies conjugated to biotin were added to the different cytokines. For development of the bond, a developer solution containing streptavidin-peroxidase, substrate and a chromogen enzyme conjugate were added. The reaction was read at 450 nm in a M2 spectrophotometer. Sample concentrations were calculated using the standard curves obtained with the recombinant cytokines and the result was expressed per µg/ml.

### Apoptosis of osteoblasts Caspase-3

To perform osteoblast apoptosis analyses, slides were deparaffinized in hot xylene at 60–65 °C and rehydrated in decreasing concentrations of ethanol, running water and distilled water. The antigenic epitopes were recovered by high temperature in 10 mM citrate solution (pH = 6.0) and the unspecific oxidation reactive sites were blocked with endogenous peroxidase at room temperature. Then, the slides were incubated overnight at 4 °C with primary antibody Caspase-3 anti-rabbit polyclonal IgG antibody (H-277): sc-7148, Sta. Cruz Biotechnology, Ca, EUA) diluted (1:100) in PBS was incubated at room temperature for 60 min and after this period, the sections were washed with phosphate buffer (PBS–pH = 7.4) and incubated with secondary antibody according to the origin of the antibody primer at 37 °C. Finally, for the development, chromogen solution (DAB) and Harris' hematoxylin were used to counterstain. For each specific marker, a specific primary antibody was used with its respective secondary antibody. The dilution of the same was standardized, starting the tests with the dilutions suggested by the manufacturers, which were described in the following table: For development we used the Vectastin Abc Kit, Vector Laboratories (Sta. Cruz Biotechnology, CA, USA) or Envisionision + Dual Link System Peroxidase (DakoCytomation, CA, USA). The samples were analyzed blindly, with 5–10 fields selected in the trabecular surface region at a magnification of 400x. The density of positive cells were analyzed and were count with Software Image Pro-Plus 6.0. The result was expressed as cells/μm^2^.

### Statistical analysis

Statistical analysis was performed using the SigmaStat program (Systat Software, San Jose, CA, USA; version 11.0). Kruskal–Wallis one-way analysis of variance on ranks and an all pairwise multiple comparison procedures (Dunn’s Test) were used to test for significant differences among test groups at the *p < 0.05 level of significance. Results were expressed as the means ± SE.

## Discussion

In this study, we showed that smoking induces structural changes in bone due to increased release of pro-inflammatory mediators and consequent increase in osteoblasts apoptosis. Moreover, even in former smokers, part of these deleterious effects are also observed.

We showed histological changes in bone tissue in smokers characterized by a decrease in trabecular area, reduction in collagen type I and an increase in collagen type V. Although former smokers did not show a decrease in trabecular area and neither increase in collagen type V, they demonstrated decrease in collagen type I and changes in inflammatory cytokine levels similar to smokers.

It is interesting that the majority of former smokers, in our study, showed a long time of smoking cessation (above 10 years). In clinical practice of trauma surgeries, it has been recommended that patients must quit smoking at least 6 weeks after surgical procedure. However, some studies showed that despite Smoking cessation restores the tissue microenvironment and the inflammatory cellular functions within 4 weeks which reduces surgical site infections, it is not effective to avoid healing complications^22^.

The bone healing complications after surgery in both smokers and former smokers have been attributed mostly to the impairment in cells activity leading alterations in collagen metabolism. The worsening in bone cells activity occurs due the increased levels of pro-inflammatory cytokines induced by smoking exposure^22^.

Increased levels of IL-1β, IL-6 and TNF-α were observed in bone tissue homogenates. These cytokines effects in stimulation of osteoclast formation and activation and consequent increase in bone resorption have been described in literature^23–25^. Moreover, TNF-α is recognized by the induction of osteoblasts apoptosis^26^. Alikhani et al. showed that the increase in osteoblasts apoptosis was associated to an increase in TNF-α levels in human primary osteoblastic cells^27^.

Apoptosis is a mechanism that regulates cells death, contributing to all self-renewing tissues^28^ and smoking is recognized by the induction of cells apoptosis in different body tissues^29–31^. Considering that the balance between osteoclast and osteoblast proliferation and apoptosis determine the bone turn over^26^, the decrease in type I collagen, observed in our study, could be in part attributed to the increase in osteoblasts apoptosis.

It is important to note that although both smokers and former smokers showed increase in osteoblasts apoptosis with concomitant decrease in type I collagen deposition, only smokers showed reduction in trabecular area with concomitant increase in collagen type V. Also, only smokers showed reduction in OPG levels in tissue homogenate, suggesting higher osteoclast activation and increased bone resorption. Considering that type V collagen fibers regulate the diameter of heterotypic fibrils and that the increase in this collagen type predispose to the deposition of fibers with smaller diameters^32–35^ our results suggest that smokers showed higher bone fragility compared with other groups.

## Conclusions

Smoking induces a worsening in bone matrix composition by increase in inflammatory mediators that leads changes in bone cells physiology. The increase in in osteoblasts apoptosis was found as playing a pivotal role in these events. These results reinforce clinical evidence that smokers showed bone fragility associated with higher incidence of bone fracture events.

## Data Availability

The dataset used during the current study is available from the corresponding author, Lopes F. D. T. Q. S. (fernandadtqsl@gmail.com), on reasonable request.
